# Successful Emergency Removal of a Titanium Penile Constriction Ring Using Blood Aspiration and Nonpowered Bolt Cutters

**DOI:** 10.7759/cureus.104026

**Published:** 2026-02-21

**Authors:** Gabriella F Weston, Bradley M Golden

**Affiliations:** 1 Emergency Medicine, Augusta University Medical College of Georgia, Augusta, USA

**Keywords:** bolt cutters, emergency ring removal, penile constriction injury, penile strangulation, procedural sedation and analgesia, titanium ring entrapment, urologic emergency

## Abstract

Penile constriction injuries are rare urologic emergencies that can progress to ischemia if not promptly relieved. Metallic devices composed of titanium present unique challenges due to their strength and resistance to standard emergency department cutting tools. We report a 52-year-old male patient who presented with acute penile pain, swelling, and discoloration after a titanium wedding ring became entrapped at the penile base for four hours. Conventional ring cutters were ineffective, and no powered surgical tools were immediately available. The patient underwent ketamine procedural sedation and dorsal penile nerve block. Approximately 50 mL of blood was aspirated to reduce engorgement and allow safe placement of protective barriers. A nonpowered 24-inch bolt cutter obtained from hospital facilities was used to fracture the titanium ring without tissue injury. Penile color and edema improved immediately. The patient was observed, successfully voided, and was discharged with urologic follow-up. This case highlights a practical approach for emergent removal of titanium penile constriction rings when standard equipment fails, emphasizing multidisciplinary coordination and staff safety precautions.

## Introduction

Penile constriction injuries represent uncommon but potentially severe urologic emergencies. Entrapped constriction devices may cause venous outflow obstruction, progressive edema, ischemia, and, in severe cases, tissue necrosis or gangrene. Rapid intervention is essential to preserve tissue viability and prevent long-term complications.

Penile ring entrapment has been described as a true urologic emergency, and structured grading systems and management approaches have been proposed to guide timely removal and reduce morbidity [1]. In prolonged entrapment scenarios, case reports and literature reviews emphasize the importance of multidisciplinary coordination and timely access to appropriate tools when conventional instruments fail [2].

Metallic rings composed of titanium present a unique challenge in the emergency setting. Titanium’s strength, resilience, and resistance to deformation often exceed the capabilities of standard emergency department ring cutters and may generate heat or sparks during fracture, necessitating alternative tools and procedural adaptations [3]. Nonsurgical and extrication-focused reports describe a range of decompression and removal strategies when conventional techniques are unsuccessful, particularly with hardened metallic devices [4-6].

We present a case of successful titanium penile ring removal using aspiration decompression and nonpowered bolt cutters.

## Case presentation

A 52-year-old male patient with no significant past medical history presented to the emergency department with penile pain and progressive swelling after placing a titanium wedding ring around the base of his penis approximately four hours prior to arrival. The patient reported increasing discomfort and inability to remove the ring manually.

**Figure 1:**
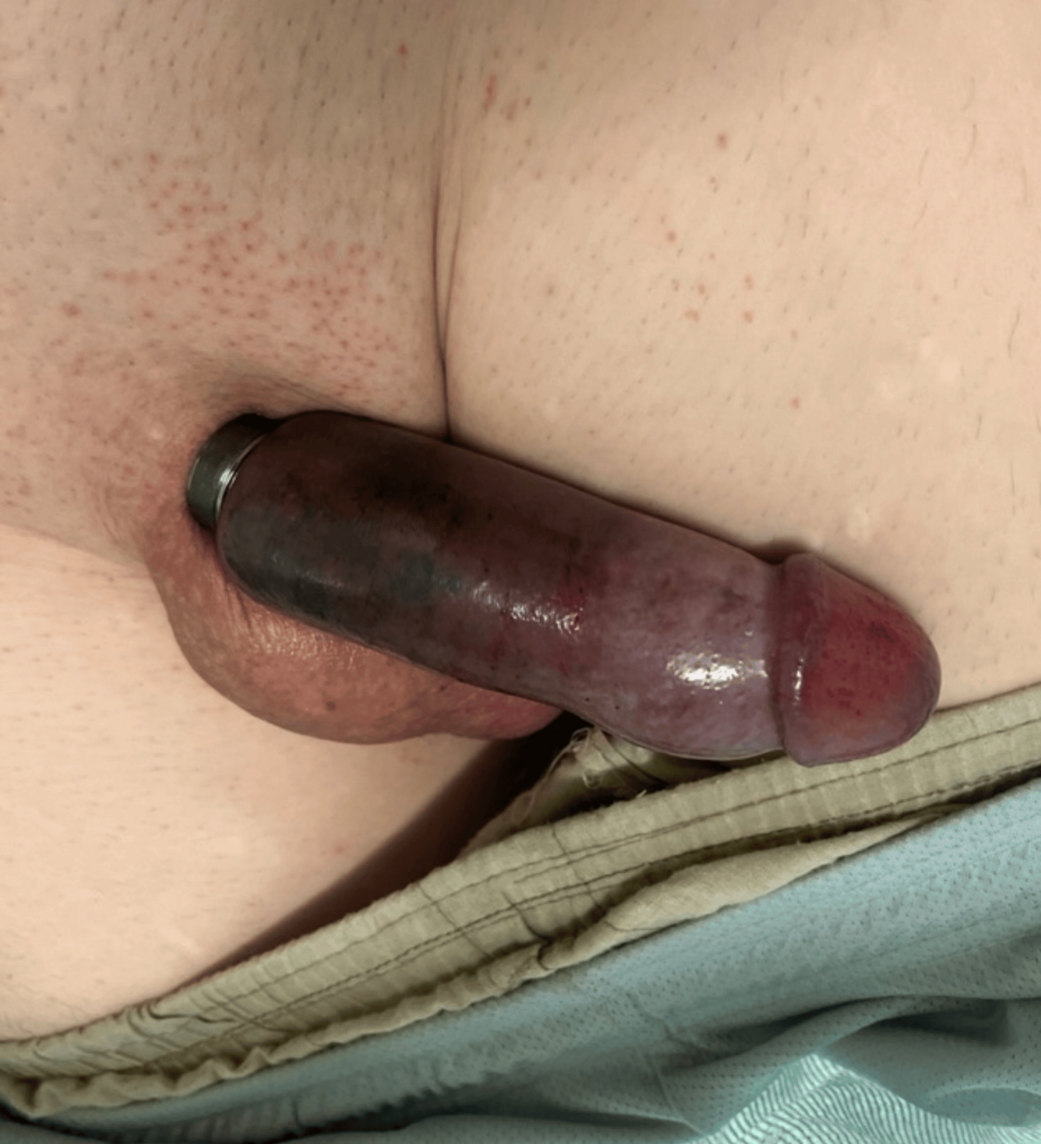
Penile edema and discoloration caused by titanium ring entrapment at the penile base prior to device removal.

On examination, a thick metallic ring was tightly constricting the penile base with marked distal edema, venous congestion, and dusky discoloration (Figure 1). Sensation was intact, and there was no active bleeding, but concern existed for worsening vascular compromise with prolonged entrapment.

Urology was urgently consulted. Initial conservative measures included application of ice packs and attempts at lubrication and manual removal. These efforts were unsuccessful due to severe swelling and the rigidity of the titanium ring.

Standard emergency department ring-cutting equipment was ineffective, as the available manual ring cutter was rated only for softer metals such as gold or silver. A ring cracker could not be positioned due to limited space between the ring and edematous tissue. Orthopedic surgery and operating room personnel were contacted but could not provide powered cutting tools suitable for titanium. Ultimately, hospital facilities supplied a nonpowered 24-inch bolt cutter as the only available instrument capable of addressing the device.

Given significant pain and the need for urgent removal, the patient underwent procedural sedation with ketamine. A dorsal penile nerve block was performed for additional analgesia. To reduce penile engorgement and improve access, approximately 50 mL of blood was aspirated using sterile technique, resulting in partial decompression.

A sterile S-shaped retractor and folded operating room towel were placed between the ring and penile skin to prevent laceration. Staff wore eye protection due to the risk of sparks or metallic fragments. The bolt cutter was carefully applied, and the titanium ring fractured abruptly, producing a visible spark and small shrapnel fragments. No tissue injury occurred.

Immediately following removal, penile color improved and swelling decreased significantly. The patient was monitored in the emergency department for several hours, successfully completed a spontaneous voiding trial, and was discharged with outpatient urology follow-up and strict return precautions.

## Discussion

Penile constriction injuries are rare but require urgent intervention to prevent ischemic injury. The underlying pathophysiology typically begins with venous outflow obstruction, leading to progressive swelling, impaired arterial inflow, and eventual tissue necrosis if prolonged.

Penile ring entrapment has been described as a true urologic emergency, and Dawood et al. proposed grading and management strategies emphasizing early removal to avoid severe complications [1]. Delayed intervention increases the risk of urethral injury, tissue necrosis, erectile dysfunction, and, in extreme cases, penile loss.

Several extrication techniques have been described, including string methods, aspiration decompression, dental saws, orthopedic tools, and high-speed rotary devices [5,6]. Rahmita et al. reported favorable outcomes using effective nonsurgical management strategies for metallic penile entrapment, emphasizing early decompression and careful mechanical removal [4].

Titanium presents unique technical challenges due to its high tensile strength, resilience, and spark- and heat-generating properties during cutting or fracture, making removal with standard emergency department equipment difficult [3]. Clinicians may need to coordinate with multiple services, including urology, orthopedics, the operating room, hospital facilities, or even local fire departments to obtain appropriate removal tools.

In prolonged entrapment scenarios, Puspaningrat et al. highlighted the importance of multidisciplinary coordination and timely access to appropriate equipment when conventional instruments fail [2]. In the present case, aspiration of approximately 50 mL of blood reduced penile engorgement, improving access and maneuverability. Procedural sedation and dorsal penile nerve block provided patient comfort and procedural safety. Protective barriers were essential to prevent tissue injury during device fracture.

The use of nonpowered bolt cutters successfully shattered the titanium ring without causing penile laceration or thermal injury. However, titanium fracture produced sparks and shrapnel, underscoring the importance of eye protection and shielding of surrounding tissue.

While topical ice may reduce swelling, phenylephrine administration is contraindicated in penile strangulation injuries because vasoconstriction may worsen ischemia in the setting of mechanical venous outflow obstruction.

This case contributes to the growing literature supporting decompression-assisted removal strategies and highlights a practical approach when specialized urologic equipment is unavailable.

## Conclusions

Entrapped titanium penile rings represent a time-sensitive urologic emergency that may not be manageable with standard emergency department ring cutters. When specialized surgical tools are unavailable, nonpowered bolt cutters may provide a safe and effective alternative. Adjunctive aspiration decompression, procedural sedation, nerve block, and tissue shielding can facilitate removal while minimizing complications. Emergency physicians should be prepared to involve multidisciplinary and nontraditional hospital resources and ensure appropriate safety precautions during device fracture.
